# Nutritional Supplementation in Tuberculosis Treatment: A Mixed Methods Study of Clinical Outcomes and Patient Perceptions in Jamnagar, India

**DOI:** 10.7759/cureus.70300

**Published:** 2024-09-27

**Authors:** Viral Shah, Yogesh Murugan, Shubham S Patel, Nidhi S Trivedi, Dhiren Pithadiya, Naresh Makwana, Dipesh Parmar

**Affiliations:** 1 Community and Family Medicine, Shri Meghji Pethraj (MP) Shah Government Medical College, Jamnagar, IND; 2 Community and Family Medicine, Shri Meghji Pethraj (MP) Shah Government Medical College, Guru Gobind Singh Government Hospital, Jamnagar, IND; 3 Community Medicine, Shri Meghji Pethraj (MP) Shah Government Medical College, Jamnagar, IND; 4 Respiratory Medicine, District TB Centre, Jamnagar, IND

**Keywords:** india, mixed-methods, nutritional supplementation, patient experiences, treatment outcomes, tuberculosis

## Abstract

Background: Tuberculosis (TB) remains a significant global health challenge, with malnutrition being a key risk factor for poor outcomes. This study aimed to evaluate the impact of nutritional supplementation on treatment outcomes in drug-sensitive pulmonary tuberculosis (DS-PTB) patients and explore patient perspectives on nutrition during TB treatment.

Methods: We conducted a mixed methods study in the Jamnagar district of Gujarat, India. The quantitative component was a retrospective cohort study comparing 645 DS-PTB patients who received nutritional supplements with 645 patients who did not. The primary outcomes were cure rates, mortality, and weight gain. Qualitative data were collected through in-depth interviews of 240 patients to explore their experiences and perceptions regarding nutrition during TB treatment.

Results: Patients receiving nutritional supplements had significantly higher cure rates (482/645, n=74.7% vs 328/645, n=50.9%, OR: 2.86, 95% CI: 2.26-3.61, p<0.001) and lower mortality (7/645, n=1.1% vs 37/645, n=5.7%, OR: 0.18, 95% CI: 0.08-0.41, p<0.001) compared to the non-supplemented group. The group that received nutritional supplementation showed greater weight gain over six months (6.5 kg vs 3.1 kg, p<0.001). Qualitative findings revealed that patients who received nutritional supplementation reported improved appetite, increased energy, and faster symptom resolution while control group participants faced financial constraints and reduced appetite as barriers to adequate nutrition.

Conclusion: Nutritional supplementation significantly improved treatment outcomes in DS-PTB patients, including higher cure rates, reduced mortality, and enhanced weight gain. Patient perspectives highlighted the multifaceted impact of nutritional support. These findings suggest that integrating nutritional supplementation into standard TB care could substantially improve patient outcomes and experiences.

## Introduction

Tuberculosis (TB) remains a global health challenge, and India bears a disproportionate burden of the disease. The pooled TBI prevalence for India based on the community-based cohort studies was estimated at 41% (95% confidence interval {CI} 29.5-52.6%) irrespective of the risk of acquiring it, while the estimation was 36% (95% CI: 28-45%) prevalence observed among the general population excluding high-risk groups [1]. India has the largest tuberculosis burden, but the all-age prevalence in 2021 ranged from 747/100,000 in Delhi to 137/100,000 in Gujarat [2-4].

Malnutrition and micronutrient deficiencies are widely prevalent in TB patients and associated with adverse outcomes [5]. Nutritional supplementation has shown promise in observational studies by promoting weight gain and faster sputum conversion [6]. However, data from trials on clinical benefits are sparse, with few studies adequately powered to assess the impact on mortality or treatment success rates [7].

Treatment outcomes in multidrug-resistant (MDR) TB remain poor in resource-limited settings due to factors like suboptimal drug regimens, high rates of loss to follow-up, and lack of ancillary support. Almost 50% of MDR-TB patients experience permanent or temporary hearing loss from the aminoglycosides used [8]. Gastrointestinal side effects are also common. Ensuring adequate calorie and micronutrient intake can help patients better tolerate these toxic medications.

At the same time, achieving improved nutrition may be challenging in TB patients, who often suffer from anorexia, nausea, and malabsorption [9]. This calls for innovative meal plans and culturally appropriate supplements that can help meet daily requirements despite poor appetite and intestinal dysfunction. Our study provides field evidence supporting the WHO recommendation for the inclusion of a trained dietitian in MDR-TB management teams [10].

An additional benefit of nutritional rehabilitation is facilitating recovery of muscle mass and reversal of wasting. This helps restore functional capacity and enables patients to resume work and livelihoods sooner [11]. Optimal nutrition is also essential for supporting bone health, which can be compromised by medications like fluoroquinolones used in MDR-TB treatment [12]. Our significant findings on reduced morality can inform health policies on integrating nutritional supplementation in national DR-TB programs. This investment could alleviate the economic burden incurred by households affected by TB [13].

In this context, we conducted a retrospective cohort study in DS-TB patients to evaluate if providing nutritional supplements as adjunct therapy improved definitive clinical outcomes such as cure and mortality over and above standard TB treatment alone. Also, the qualitative study aimed to address this gap by exploring the experiences, perceptions, and suggestions of TB patients who received nutritional supplementation during their treatment, as well as those who did not receive supplementation. We hypothesized that ensuring adequate nutrition through the provision of supplements helps strengthen immune function, counters inflammation, and improves absorption of anti-TB drugs. This can enhance the host’s resilience and capacity to suppress and eliminate the infection more effectively.

## Materials and methods

Study design and duration

We undertook sequential explanatory mixed methods research among patients of pulmonary tuberculosis in the Jamnagar district of Gujarat. We conducted a retrospective cohort study to determine the association of nutrition with weight gain and treatment outcomes of pulmonary tuberculosis (PTB). This was followed by qualitative, in-depth interviews. The purpose of in-depth interviews was to explore the patient's perspectives on the importance of nutrition in TB and its effect on collaborative TB-nutritional activities. The qualitative component of the study was founded on the constructivist paradigm of knowledge acquisition. According to the constructivist paradigm, individuals construct their perception of the world via their experiences and interactions with others, implying that knowledge is continually evolving and reconstructed as people engage with new experiences and viewpoints. The purpose of this approach was to acquire a thorough understanding of the study participants’ subjective experiences and perspectives. A descriptive design was used to describe the codes and categories generated based on the in-depth interviews. The data for the retrospective cohort study were collected from February to March 2024, and parallel in-depth interviews were conducted from January to May 2024. The study follows the criteria for reporting cohort and qualitative research.

Study setting

The Jamnagar district of Gujarat state comprises around 1.4 million people as per the census 2011. This study was conducted in the Jamnagar district and Jamnagar Municipal Corporation with financial support from Nayara Energy Ltd., Jamnagar, India, and the District Tuberculosis Center (DTC), Jamnagar, India.

For operationalization of the National Tuberculosis Elimination Program (NTEP), the Jamnagar district and the municipal corporation are divided into the eight tuberculosis units (TUs) under District Tuberculosis Centre (DTC) - Jamnagar Urban-1, Jamnagar Urban-2, Jamnagar Rural, Dhrol, Jodiya, Jamjodhpur, Kalavad, and Lalpur - which provide monthly follow-up and drug strips to TB patients. With the financial support of Nayara Energy Ltd. (Nikshay Mitra) and the assistance of the senior treatment supervisor (STS) from all tuberculosis units (TUs) at the Tunder District Tuberculosis Centre (DTC), nutritional kits (one kit per month) are provided to TB patients. A total of 600 nutrition kits in the year 2021 and 800 kits in the year 2022 were distributed among selected TB patients. Since 2023, all TB patients registered in DTC receive nutritional kits every month. The contents and calories are mentioned in Table 1.

**Table 1:** Nutritional kit contents with their respective calories. ^a^As mentioned on the label. ^b^As per the National Institute of Nutrition, 2017 (Indian food composition table).

Content	Quantity	Total energy^a^	Total protein
Tirupati groundnut oil	1 L (950 g)	900 kcal	-
Ashirwad multigrain atta	10 kg (2 packets of 5 kg)	33,600 kcal	1440 g^a^
Daawat Rozana basmati rice	3 kg (3 packets of 1 kg)	10,440 kcal	246 g^a^
Jaggery	1 kg	3,850 kcal	4 g^a^
Chickpeas	1 kg	3,780 kcal	190 g^b^
Moong dal	1 kg	3,470 kcal	220 g^b^

Study population

Quantitative

We included all newly diagnosed drug-sensitive pulmonary tuberculosis patients >18 years who received nutritional kits, notified under the TB program from January 2021 to December 2023 for nutritional group (cases). We chose a matched control group (age and gender matching) in 1:1 proportion following the same criteria except for received nutritional kits. We excluded recurrent, drug-resistant PTB, extra-pulmonary TB, and those below 18 years of age. Patients with significant comorbidities that could substantially impact TB treatment outcomes or nutritional status, including but not limited to advanced HIV disease (CD4 count <200 cells/μL), severe liver disease (Child-Pugh class C), severe kidney disease (eGFR <30 mL/min/1.73 m²), active malignancy, and severe heart failure (New York Heart Association {NYHA} class III or IV).

Patients with controlled diabetes mellitus and well-managed HIV (on antiretroviral therapy {ART} with CD4 count >200 cells/μL) were included in the study, as these represent common comorbidities in TB patients. Their status was recorded and considered in the analysis.

Qualitative

Patients who received more than three nutritional kits were taken as cases and those who did not receive nutritional kits were taken as control, both groups were included in in-depth interviews. Important points were taken down during the interviews. The interviews were conducted until the sample size we calculated was reached. A total of 240 in-depth interviews were carried out.

Sample size and sampling technique

Quantitative

A universal sampling method was used. All patients registered under the District Tuberculosis Centre of Jamnagar District and Corporation as per Nikshay TB portal from January 2021 to December 2023 were included. Our study included a total of 1,290 participants, with 645 in each group (nutritional supplementation and control). This sample size was determined based on an assumed medium effect size (Cohen's d=0.5) for our primary outcome measures. A sensitivity power analysis revealed that this sample size would provide more than 80% power to detect the expected effect size, with a two-tailed Type I error rate (α) of 0.05. Further sensitivity analyses demonstrated that our sample size maintained >80% power for detecting effect sizes as small as Cohen's d=0.35 and remained robust when accounting for our observed dropout rate (2.5% in the control group). Additionally, this sample size allowed for meaningful subgroup analyses while maintaining sufficient statistical power. By striking a balance between statistical power and feasibility, our sample size enables us to draw reliable conclusions about the impact of nutritional supplementation on TB treatment outcomes, detect smaller effect sizes than initially anticipated, and efficiently utilize available resources.

Qualitative

From each tuberculosis unit (TU), 30 patients were chosen for in-depth interviews to explore the perception of patients towards nutrition kits who attained a minimum of 18 years of age and registered under the Nikshay TB portal from January 2021 to December 2023. Interviews of 15 participants who received a minimum of three kits (cases) and 15 participants who did not receive any kit (controls) were taken from each TU. Thus, in eight TUs total of 240 patients were interviewed.

Study variables (quantitative)

Outcome Variable

The national TB program defines treatment outcomes for TB patients put on treatment. Treatment success is defined as when patients test negative on sputum/culture at the end of treatment (termed "cured") or have completed treatment without any evidence of clinical or radiological deterioration (defined as "treatment completed"). Unfavorable TB treatment outcomes are defined when patients stop treatment for at least one consecutive month (categorized as "lost to follow-up"), test positive on sputum/culture at the end of treatment (categorized as "treatment failure"), or die while on treatment (categorized as "died").

Exposure Variable

The exposure variable was dichotomous. The exposed group consisted of nutritional group patients who received nutritional kits, while the unexposed group consisted of TB patients who did not receive nutritional kits.

Confounding Variables

We considered several potential confounding factors in our analysis, including demographic, clinical, and socioeconomic variables. These included demographic factors, such as age and gender; clinical factors, such as diabetes status, HIV status, and weight (BMI) at baseline; behavioral factors, such as tobacco use and alcohol consumption; and socioeconomic factors, such as educational level and household income.

Due to the retrospective nature of our study, we were limited by the availability of data in existing records. While we were able to match cases and controls on age and gender, we acknowledge that we lacked comprehensive data on socioeconomic status and education level for all participants.

Data collection

Quantitative

The DTC located in Jamnagar district was visited to collect data. The Nikshay portal was used to download data in Excel sheet format. Nikshay online portal was launched in 2017, which maintains records of notified TB patients of the whole nation. The sheet was collected with the help of the program coordinator. An Excel document for the data from January 2021 to December 2023 was taken, which contains all patient's records (such as Niskhay ID, socio-demographic details, TU, habits, baseline characteristics, etc.). A separate sheet of patients who received nutritional kits has been available to STS and the program coordinator since the initiation of this project of nutrition kit distribution. All data were collected by investigators. A total of 5,934 patients were registered in a span of three years and 3,273 were excluded as they did not meet the inclusion criteria. Of the 2,661 patients, a total of 700 received nutritional kits. Baseline weight and treatment outcome data were not available for 55 patients, who were excluded from the study. Therefore, 645 patients were included in the final analysis of the nutritional arm as cases. Out of 1,961 TB patients who did not receive nutritional kits, 645 age and gender-matched TB patients were selected in the control arm to achieve a case-control ratio of 1:1. As we did TU wise age and gender matching for case and control, it was difficult to achieve 1:2 ratio due to lesser proportion of controls in some TUs.

Qualitative

A day training programme was conducted for postgraduate students involved in the study. In-depth interviews were done by postgraduate students of the community medicine department who are qualified and trained for conducting qualitative in-depth interviews. It was community-based house-to-house in-depth interviews of selected participants for both cases (who received a minimum of three nutrition kits) and the control group (who did not receive any nutrition kit). There was no other person (third person) present during the interviews and lasted for 45-60 minutes. There were no follow-up interviews or field notes taken to collect more data.

Statistical analysis

For the quantitative component, we employed a range of statistical methods to analyze our data. Descriptive statistics were used to summarize baseline characteristics of both the nutritional and non-nutritional groups, with continuous variables expressed as means±standard deviations and categorical variables as frequencies and percentages. We conducted baseline comparisons between the two groups using independent t-tests for continuous variables and chi-square tests for categorical variables. Treatment outcomes were compared between groups using chi-square tests, and we calculated odds ratios (OR) with 95% confidence intervals (CI) to quantify the association between nutritional supplementation and various treatment outcomes. To analyze weight changes over time, we used repeated measures ANOVA, with time (baseline, three months, and six months) as the within-subjects factor and group (nutritional vs non-nutritional) as the between-subjects factor. Post-hoc analyses were conducted using Tukey's honestly significant difference (HSD) test. We considered a p-value <0.05 as statistically significant for all analyses. All statistical analyses were performed using SPSS version 26.0 (Armonk, NY: IBM Corp.).

For the qualitative component, we employed thematic analysis using a constant comparative method. In-depth interviews were transcribed verbatim and translated into English when necessary. Two researchers independently coded the transcripts, identifying key themes and subthemes. We developed initial codes based on the research questions and emerging themes from the data, refining these through iterative discussions among the research team. ATLAS.ti software version 9 (ATLAS.ti GmbH: Berlin, Germany) was used to facilitate the coding and analysis process. Themes and subthemes were identified and refined through an iterative process of coding, memo-writing, and team discussions until consensus was reached. We selected representative quotes to illustrate each theme and subtheme. To ensure trustworthiness, we conducted member checking with a subset of participants to verify the accuracy of our interpretations. Throughout the analysis process, we maintained an audit trail to document decision-making and enhance the transparency and reliability of our findings. To address the issue of missing data, we employed multiple imputation techniques to estimate missing values for participants with partial data. This method creates multiple plausible imputed datasets and combines results to account for the uncertainty around missing values.

## Results

Table 2 presents the baseline characteristics of the study participants, comparing the nutritional group (n=645) with the non-nutritional group (n=645). The table shows that the two groups were well-matched at baseline, with no statistically significant differences in key demographic and clinical variables. The mean age was similar in both groups (42±16.7 years in the nutritional group vs 42.4±16.7 years in the non-nutritional group, p=0.664). The gender distribution was nearly identical, with approximately 385/645 (59.69%) males in the nutritional group and 386/645 (59.84%) males in the non-nutritional group (p=0.995). Diabetes status was also comparable, with slightly fewer diabetics in the nutritional group (41/641, 6.40% vs 46/640, n=7.19%, p=0.326). HIV status was also comparable, with slightly fewer HIV-positive individuals in the non-nutritional group (7/642, 1.09% vs 12/640, 1.88%, p=0.176).

**Table 2:** Characteristics of participants in both groups. ^a^The diabetes status of nine participants and the HIV status of eight participants were unknown and therefore excluded from the analysis. ^b^Sixteen participants did not declare their tobacco intake habit and 36 participants did not declare their alcohol intake habit, were excluded from the analysis. P-value <0.05 was considered statistically significant.

Characteristic		Cases (received nutrition kit) (n=645) n (%)	Control (not received nutrition kit) (n=645) n (%)	Total	p-Value
Age	Mean±SD	42±16.7	42.4±16.7	-	0.664
Baseline weight	Mean±SD	46.6±10.3	47.7±11.6	-	0.072
Gender	Female	260 (40.31%)	259 (40.16%)	519 (40.2%)	0.995
	Male	385 (59.69%)	386 (59.84%)	771 (59.8%)	
Tuberculosis units (TUs)	Jamnagar Rural	136 (21.09%)	136 (21.09%)	413 (64.03%)	1.00
	Jamnagar Urban-1	123 (19.07%)	123 (19.07%)	105 (16.28%)	
	Jamnagar Urban-2	122 (18.91%)	122 (18.91%)	244 (37.83%)	
	Dhrol	50 (7.75%)	50 (7.75%)	100 (15.50%)	
	Jodiya	48 (7.44%)	48 (7.44%)	96 (15.00%)	
	Jamjodhpur	65 (10.08%)	65 (10.08%)	130 (20.16%)	
	Lalpur	53 (8.22%)	53 (8.22%)	106 (16.43%)	
	Kalavad	48 (7.44%)	48 (7.44%)	96 (14.88%)	
Diabetes (n=1281)^a^	No	594 (92.81%)	600 (93.60%)	1194 (93.21%)	0.326
	Yes	46 (7.19%)	41 (6.40%)	87 (6.79%)	
HIV (n=1282)^a^	Non-Reactive	628 (98.13%)	635 (98.91%)	1263 (98.52%)	0.176
	Reactive	12 (1.88%)	7 (1.09%)	19 (1.48%)	
Alcohol (n=1254)^b^	No	617 (98.90%)	618 (98.10%)	1235 (98.50%)	0.183
	Yes	7 (1.10%)	12 (1.90%)	19 (1.50%)	
Tobacco user (n=1274)^b^	No	483 (76.06%)	482 (75.43%)	965 (75.75%)	0.422
	Yes	152 (23.94%)	157 (24.57%)	309 (24.25%)	

Table 3 presents the treatment outcomes for both study groups, along with odds ratios (OR) to quantify the differences. The results show significant improvements in several key outcomes for the nutritional group. The cure rate was substantially higher in the nutritional group (482/645, 74.7% vs 328/645, 50.9%; OR: 2.86; 95% CI: 2.26-3.61; p<0.001), indicating that patients receiving nutritional supplementation were nearly three times more likely to be cured compared to those who didn't. Conversely, the rate of treatment completion without a confirmed cure was lower in the nutritional group (142/645, 22.0% vs 243/645, 37.7%; OR: 0.47; 95% CI: 0.37-0.59; p<0.001), likely because more patients in this group achieved confirmed cure. Importantly, mortality was significantly reduced in the nutritional group (7/645, 1.1% vs 37/645, 5.7%; OR: 0.18; 95% CI: 0.08-0.41; p<0.001), suggesting that nutritional supplementation may have a protective effect against death during TB treatment. The nutritional group also had lower rates of loss to follow-up (5/645, 0.8% vs 16/645, 2.5%; OR: 0.31; 95% CI: 0.11-0.85; p=0.022) and treatment regimen changes (5/645, 0.8% vs 18/645, 2.8%; OR: 0.27; 95% CI: 0.10-0.74; p=0.011). There was no significant difference in treatment failure rates between the groups (4/645, 0.6% vs 3/645, 0.5%; OR 1.33; 95% CI: 0.30-5.98; p=0.706). Overall favorable outcome (cure rate and treatment completed) was significantly higher in the group receiving nutritional kits as compared to the non-nutrition group (624/645, 96.74% vs 571/645, 88.53%; OR: 3.85; 95% CI: 2.34-6.33; p<0.0001). These results strongly suggest that nutritional supplementation improves treatment outcomes in TB patients, particularly in terms of cure rates and mortality reduction.

**Table 3:** Treatment outcome in cases (received nutrition kit) vs control (not received nutrition). ^a^For the cases (received nutrition kit) group, n=624 (96.74%). ^b^For the control (not received nutrition kit) group, n=571 (88.53%). ^c^For the cases (received nutrition kit) group, n=21 (3.26%). ^d^For the control (not received nutrition kit) group, n=74 (11.47%). ^e^Odds ratio (OR) and confidence interval (CI) calculated for favorable and unfavorable outcomes in both arms. ^f^P-value <0.05 was considered statistically significant.

The outcome of TB patients		Cases (received nutrition kit) (n=645) n (%)	Control (not received nutrition kit) (n=645) n (%)	Total (n=1,290) n (%)	OR (95% CI)	p-Value
Favorable outcome (n=1,195)	Cured	482 (74.7%)	328 (50.9%)	810 (62.79%)	2.86 (2.26-3.61)	<0.001^f^
	Treatment completed	142 (22.0%)	243 (37.7%)	385 (29.84%)	0.47 (0.37-0.59)	<0.001^f^
		624 (96.74%)^a^	571 (88.53%)^b^	1,195 (92.64%)	3.85 (2.34-6.33)^e^	<0.0001^f^
Unfavorable outcome (n=95)	Treatment failed	04 (0.6%)	03 (0.5%)	07 (0.54%)	1.33 (0.30-5.98)	0.706
	Died	07 (1.1%)	37 (5.7%)	44 (3.41%)	0.18 (0.08-0.41)	<0.001^f^
	Lost to follow-up	05 (0.8%)	16 (2.5%)	21 (1.63%)	0.31 (0.11-0.85)	0.022^f^
	Treatment regimen changed	05 (0.8%)	18 (2.8%)	23 (1.78%)	0.27 (0.10-0.74)	0.011^f^
		21 (3.26%)^c^	74 (11.47%)^d^	95 (7.36%)	3.85 (2.34-6.33)^e^	<0.0001^f^

Table 4 presents weight difference data which shows the progression of weight gain in both groups over time. At baseline, there was no significant difference between the groups (46.6±10.3 kg vs 47.7±11.6 kg, p=0.072). However, by three months, the nutritional group had gained significantly more weight (50.2±10.8 kg vs 48.9±11.9 kg, p=0.040). This difference became even more pronounced at six months (53.1±11.2 kg vs 50.8±12.1 kg, p<0.0004). Within-group analysis shows that both groups experienced significant weight gain over time (p<0.001 for the nutritional group, p=0.002 for the non-nutritional group), but the gain was more substantial in the nutritional group.

**Table 4:** Periodic weight gain in cases (received nutrition kit) vs control (not received nutrition kit). ^a^P-value <0.05 was considered statistically significant. Values are presented as mean±standard deviation. All weight measurements are in kilograms (kg). Between-group p-values compare groups at each time point. Within-group p-values compare across time points within each group. Differences within each group across time points were given based on Tukey's HSD test. Tukey HSD q-values: p<0.05. For Tukey HSD, different letters within a column indicate significant differences (p<0.05) between time points. Tukey's HSD: Tukey's honestly significant difference

Timeline	Cases (received nutrition kits) weight (kg) (mean±SD)	Cases periodic weight gain (kg)	Controls (not-received nutrition kits) weight (kg) (mean±SD)	Controls periodic weight gain (kg)	Between-group difference (kg)	Between-group p-value	Cases within-group p-value	Controls within-group p-value
Baseline weight	46.6±10.3	-	47.7±11.6	-	-1.1	0.091	-	-
3 months	50.2±10.8	3.6	48.9±11.9	1.2	1.3	0.043^a^	<0.0001^a^	0.067
6 months	53.1±11.2	6.5	50.8±12.1	3.1	3.7	<0.001^a^	<0.0001^a^	0.0001^a^
Overall within-group p-value	-	-	-	-	-	-	<0.0001^a^	0.002^a^
3 months vs 6 months	-	2.9	-	1.9	-	-	<0.0001^a^	0.0045^a^

The qualitative data presented in Table 5 provide insights into nutritional arm participants' experiences and perceptions. In the nutritional arm, themes included perceptions of nutritional supplements (with subthemes on tolerability, perceived benefits, and side effects), impact on TB outcomes (improved appetite, increased strength, and faster symptom resolution), barriers and facilitators to adherence, and stigma associated with TB and malnutrition. Participants generally reported positive experiences with the supplements, noting improvements in appetite, energy, and weight gain. However, some faced challenges with accessibility and financial constraints.

**Table 5:** Themes and illustrative quotes from nutritional arm participants (overall).

Theme	Subtheme	Illustrative quote
Perceptions of nutritional supplements	Tolerability	"The supplements had a pleasant taste and were easy to swallow."
	Perceived benefits	"I noticed a significant improvement in my appetite and overall energy levels."
	Side effects	"While the supplements were effective, the taste was a bit off-putting for me."
Impact on TB outcomes	Improved appetite and weight gain	"I experienced noticeable weight gain, which was encouraging during my treatment."
	Increased strength and energy	"The extra nutrition provided me with the energy to carry out daily activities."
	Faster symptom resolution	"I observed a quicker resolution of my TB symptoms with the nutritional supplementation."
Barriers and facilitators to adherence	Accessibility	"The cost of the supplements was manageable, but accessibility was sometimes a challenge."
	Social support	"Family reminders helped maintain a consistent supplement routine."
	Financial constraints	"I faced financial constraints at times, making it challenging to afford the supplements regularly."
Stigma associated with TB and malnutrition	Internalized shame	"I felt so ashamed having TB at my age like I had done something wrong."
	Feared judgment	"I hid my TB diagnosis from my coworkers, worried they would treat me differently."
	Stigmatizing attitudes	"Some people avoided me once they found out I had TB."
	Stigma barrier to adherence	"I was hesitant to pick up my supplements from the clinic at first, worried others may judge me for having TB."

Table 6 shows the qualitative data summary of the control arm, with themes centered around barriers to adequate nutritional intake (financial limitations, physical access issues, reduced appetite), perspectives on the importance of nutrition (linking it to symptom severity and viewing it as an unmet need), suggestions for nutritional support (such as food rations and nutrition education), and societal stigma surrounding TB. Control group participants expressed beliefs that better nutrition could have improved their treatment experience and outcomes. Both groups reported experiences of stigma related to TB, which affected their treatment adherence and social interactions. These qualitative findings provide context to the quantitative results, highlighting the multifaceted impact of nutritional supplementation and the challenges faced by TB patients, particularly those without additional nutritional support.

**Table 6:** Themes and illustrative quotes from control arm participants (overall).

Theme	Subtheme	Illustrative quote
Barriers to adequate nutritional intake	Financial limitations	"I couldn't afford a proper diet, and my financial situation affected my ability to buy nutritious food."
	Physical access	"Due to my weakened state, I struggled with going out to purchase food."
	Reduced appetite	"I often had a reduced appetite, and some foods were hard to tolerate during my treatment."
Perspectives on the importance of nutrition	Link to symptom severity	"I believe better nutrition could have lessened the severity of my TB symptoms."
	Belief in supplementation benefits	"I think providing nutritional supplements would be beneficial for TB patients like me."
	Unmet need	"Nutrition felt like a neglected aspect of my treatment, and I felt it was an unmet need."
Suggestions for nutritional support	Food rations	"Having access to food rations or baskets during treatment would have eased my situation."
	Micronutrient supplements	"Supplementing with essential micronutrients could be a valuable addition to TB care."
	Nutrition education	"I believe more counseling on what foods to eat during TB treatment is crucial for patients."
Societal stigma surrounding TB	Negative reactions	"People assumed only dirty or poor people get TB."
	Stereotypes	"I stopped participating in community events once I got diagnosed."
	Judgment and misconceptions	"The stigma made me hesitate to go to the clinic at first."

## Discussion

Our study provides compelling evidence for the significant impact of nutritional supplementation on treatment outcomes in patients with drug-sensitive pulmonary tuberculosis (DS-PTB). The results demonstrate substantial improvements in cure rates, reduced mortality, and enhanced weight gain among patients receiving nutritional supplements compared to those who did not.

The markedly higher cure rate in the nutritional group (74.7% vs 50.9%; OR: 2.86; 95% CI: 2.26-3.61; p<0.001) aligns with previous studies that have suggested the potential benefits of nutritional support in TB treatment. For instance, Franke et al. found that nutritional supplementation improved sputum conversion rates, a key indicator of treatment success [14]. Our findings extend this work by demonstrating a direct impact on cure rates, providing stronger evidence for the integration of nutritional support into standard TB care protocols.

The significant reduction in mortality observed in the nutritional group (1.1% vs 5.7%; OR: 0.18; 95% CI: 0.08-0.41; p<0.001) is particularly noteworthy. This finding resonates with a systematic review by Bhargava et al., which suggested that nutritional supplementation might reduce mortality in TB patients, although their review called for more robust evidence [15]. Our study provides this much-needed evidence, demonstrating a clear survival benefit associated with nutritional supplementation.

The enhanced weight gain observed in the nutritional group (6.5 kg vs 3.1 kg over six months, p<0.001) is consistent with the findings of a previous study, which reported improved weight gain and lean mass with nutritional supplementation in TB patients [16]. Bhargava et al. found in their study that this weight gain is clinically significant, as malnutrition is a known risk factor for poor TB outcomes and increased mortality [17].

Our qualitative findings provide crucial context to these quantitative results. The themes that emerged from the nutritional group, such as improved appetite, increased energy, and faster symptom resolution, align well with the observed clinical outcomes. These subjective improvements likely contributed to better treatment adherence, as suggested by the lower rates of loss to follow-up in the nutritional group (0.8% vs 2.5%, p=0.022).

The barriers to adequate nutrition identified in the control group, including financial limitations and reduced appetite, highlight the challenges faced by TB patients in maintaining optimal nutritional status during treatment. These findings echo those of a previous study, which identified similar nutritional challenges among TB patients [18].

The stigma associated with TB, reported by participants in both groups, remains a significant concern. This stigma can lead to delayed diagnosis, poor treatment adherence, and social isolation, as noted by a previous study [19]. Our findings suggest that nutritional supplementation programs could potentially serve as a platform for addressing stigma through education and community engagement.

While our study provides valuable insights into the impact of nutritional supplementation on TB treatment outcomes, several limitations warrant consideration. The retrospective cohort design first introduces the potential for selection bias and unmeasured confounding. Despite our efforts to match participants and control for known confounders, residual confounding from factors, such as socioeconomic status, severity of TB at baseline, or concurrent medications, cannot be ruled out. Secondly, the lack of randomization may have introduced bias in the allocation of nutritional supplementation. Thirdly, while we attempted to blind outcome assessors, complete blinding was not always possible, potentially introducing observer bias, particularly for subjective outcomes. Fourthly, our study was conducted in a single district in Gujarat, India, which may limit its generalizability to other geographic or demographic contexts. Fifthly, we were unable to assess long-term outcomes beyond the treatment period, which could provide valuable information on the sustained effects of nutritional supplementation. Lastly, our qualitative data, while informative, may not capture the full range of patient experiences due to potential selection bias in interview participation. In light of these limitations, we recommend future research directions as follows: (1) conduct randomized controlled trials to more definitively establish the causal relationship between nutritional supplementation and TB treatment outcomes; (2) implement multicenter studies across diverse geographic and socioeconomic settings to enhance generalizability; (3) incorporate long-term follow-up to assess sustained effects of nutritional intervention post-treatment; (4) utilize standardized, objective measures for all outcomes where possible; (5) conduct cost-effectiveness analyses to inform policy decisions on integrating nutritional support into TB programs; (6) explore potential mechanisms through which nutritional supplementation improves outcomes, possibly through biomarker studies; and (7) investigate the optimal composition, timing, and duration of nutritional supplementation for maximum benefit. Addressing these limitations and pursuing these research directions will significantly advance our understanding of the role of nutrition in TB treatment and inform evidence-based policies to improve patient outcomes.

## Conclusions

The present study provides an association between nutritional supplementation and TB treatment outcomes, including improved cure rates, reduced mortality, and enhanced weight gain. These findings have important implications for TB control programs, suggesting that the integration of nutritional support into standard TB care could significantly improve patient outcomes. Future prospective, randomized controlled trials are needed to further validate these findings and explore the optimal composition and duration of nutritional supplementation in TB treatment.
